# Hospital and Institutional News

**Published:** 1917-10-13

**Authors:** 


### HOSPITAL AND INSTITUTIONAL NEWS.

### A CHAIR OF TUBERCULOSIS.

The establishment at Edinburgh University of a Chair of Tuberculosis—the first at any university within the British Empire—may well become one of the landmarks in the progress of medical science in this country. It is fitting that the distinction should have been attained by Edinburgh University, for it has always been renowned for its medical faculty. It is welcome that our knowledge of the scourge which levies such a terrible toll on human life is now of sufficient extent and exact enough to be pregnant with hope for the future of humanity. Tuberculosis in its several forms is an implacable foe of the human race. A victory over it by medical science would be a triumph the whole world would hail with joy. Sir Robert Philip, who becomes the first Professor of Tuberculosis at Edinburgh, is one of the oldest living authorities on the subject. It was he who, in 1887, started the first tuberculosis dispensary the world had seen.

### SPANISH OFFICERS ON HOSPITAL SHIPS.

In pursuance of the policy mentioned in a recent Note in these columns, we observe a telegram, which lately arrived through Reuter's Agency, in which it was stated that Spanish naval officers have been placed on Italian hospital ships to protect them from the attacks of German submarines. The Spanish officers will, of course, also serve as a guarantee, if one were needed, that the hospital ships are still being used in accordance with the spirit and the letter of international law. The Italians, we believe, are the latest and last of the Allies to adopt this practice.

### THE QUEEN-MOTHER AND SALOP INFIRMARY.

Queen Alexandra has given her name in support of a scheme to establish a memorial to Mrs. Katharine Mary Harley, who, after receiving the rudiments of her training at the Salop Royal Infirmary, immediately after war was declared went to the East and was killed by a hostile shell at Monastir on March 7 last. Mrs. Harley, who offered herself to the committee of the Scottish Women's Hospitals, took out a unit to France under its direction in January 1915. She quickly established two hospitals for French wounded, and received the Croix de Guerre from General Sarrail. That this was but a proof of genuine esteem is seen in the fact that she was entrusted with the responsibility of accompanying the French Expeditionary Force to Salonika in October 1916. After returning to England to collect funds she went to Greece with a flying column of motor ambulances, till in February 1917 she was sent to help in relieving the distressed Serbians at Monastir. It was in the course of this work that she was killed. To commemorate her and her work it is hoped to endow a ward or beds at the Salop Royal Infirmary, to which she was attached by ties of interest and affection. Backed by Queen Alexandra's support and encouragement, others, including Lord French and Mrs. Millicent Garrett Fawcett, are interesting themselves in the scheme, which was started by the Shrewsbury Branch of the National Union of Women Suffrage Societies. The Katharine Mary Harley Memorial Fund should be assured of success.

### A REMODELLED COTTAGE HOSPITAL.

Among the reconstruction schemes which have survived the war is that lately completed at Halstead Cottage Hospital. This has had the advantage of a single inspiring mind in Mr. George Courtauld, the hospital's founder, who has been at pains to preserve the charming lawn and open frontage, which have not been disturbed. Had not the hospital possessed considerable ground this preservation would hardly have been possible, for the idea was to concentrate on the ground floor all rooms used in connection with the treatment of patients. Because of this happy combination of a practical aim with an artistic conscience, the additions have been made at the back and on the sides of the original building. They include a sanitary unit for each of the two wards, a nurses' duty room attached to each ward, which is seen through an observation window, while the old upstairs kitchen, converted into a bedroom, has been replaced by one on the ground floor, and to this a new scullery has been added. A new laundry has been built at the back. There is also a new ward for private patients, with a fine view of the grounds from its big window. This ward, which is being furnished, possesses an "Elizabethan" floor. In addition, the theatre has been improved and its walls painted. On the top floor are the quarters of the matron, Miss K. W. Buckingham, and the nurses' rooms, together with a sanitary unit of their own. The drainage has been thoroughly examined and in part remodelled, while space has been provided for a boiler in the basement, from which eventually the building will be warmed and hot water provided. The accommodation has been increased to twelve beds and a cot. Altogether a remodelled institution, to which Mr. Paul Waterhouse, M.A., F.R.I.B.A., gave his great experience and artistic aid.

### ILKESTON HOSPITAL.

The Duke of Rutland was re-elected president of the Ilkeston Hospital at the annual meeting of subscribers, when it was reported that, in spite of the income being the highest ever recorded, viz. £1,573, there was a deficit of £33. The gift of an *x*-ray apparatus, together with the necessary buildings, was gratefully acknowledged from Dr. and Mrs. Dobson and the members of the Ilkeston Corps of the St. John Ambulance Brigade. It was announced that the apparatus had been delivered and the building was nearly completed. The *x*-rays are now so extensively used both in the diagnosis of injury and disease and in treatment that no hospital can be considered complete that does not possess the apparatus.

### CARDIFF CENTRAL MARKET KNITTING GUILD.

Mr. Leonard D. Rea, the secretary-superintendent of King Edward VII.'s Hospital, Cardiff, in making an appeal for funds in the Press recently, mentioned that the upkeep of the institution cost £72 a day, and asked those who appreciated the work the hospital was doing to accept the responsibility of its support for one day. This novel challenge was quickly responded to by the Cardiff Central Market Knitting Guild, which is composed of about forty girls and young people engaged as assistants at the various stalls. Not only did they accept the challenge, but the self-same day a cheque for £72 was forwarded to Mr. Rea. It is worth mentioning that this band of girl workers, besides contributing such a relatively handsome sum to the hospital, have knitted and sent to the soldiers at the Front 2,050 pairs of stockings, 75 body belts, and other garments, have entertained 1,150 soldiers, and contributed £30 to local funds for the welfare of soldiers.

### NOTTINGHAM GENERAL HOSPITAL

In a report presented to the anniversary meeting of the Nottingham General Hospital, held on October 10, the monthly board stated that the Flag Day organised by the Hospital Saturday Committee brought an even more gratifying result than in 1916. It is expected that the hospital will benefit to the extent of at least £2,700, as compared with £2,678 last year. An increase of £298 was also reported in the amount received from the Hospital Saturday Fund, which amounted to £6,606. This may be regarded as a highly satisfactory sum in view of the dislocation of trade and employment through war conditions. From the Hospital Sunday Fund £1,154 was received, compared with £1,220 in 1916. The board expressed its appreciation of the generous support which enabled them, despite increased cost in every direction, to carry on fully the hospital's beneficent work. The report mentioned that the new hut to accommodate a further fifty sick and wounded soldiers has now been completed. An arrangement has been made with the local War Pensions Committee to accept disabled sailors and soldiers for treatment—in-patients at the rate of 4s. each per day, and out-patients at 7s. each for a term of two months.

### WHAT TO WRITE IN A VISITORS' BOOK.

In dealing on September 1 with the anonymous critics who by then had created a stir of resentment among the staff of Macclesfield Infirmary, we mentioned that, after hearing a deputation of nurses, the Board established a rota of house visitors. The reports of these visitors have already begun to come in, and provided that the visitors contain two or three trained observers who will make it their business to interest the public in their reports, good results should flow from the publicity which rightly is being given to them among the governors. Writing valuable notices in a visitor's book, or putting any kind of report on paper, requires a trained pen as well as a trained eye. For a happy medium must be found between mere invective and empty eulogy. If the latter prevails, the rota committee will be known as the whitewashing committee. If adverse criticism is common, there will only be another row. We would emphasise that of these two effects empty praise is as dangerous as its opposite, and, of course, is much more likely. A long experience has taught us that it is a mistake to praise "everything." The praise must be definite if it is not to be dull; and the more extensive the praise the more precise must it be. Vagueness at all costs must be avoided. A ward should not be praised as pretty, but the flowers which make it so. The same rule applies to blame, if something is to be blamed. We venture to emphasise these points, since we are as anxious as Alderman Frost himself that the rota committee should achieve its object. That object is to dispel anonymous and vague charges by a weekly report of precision and fact. Nouns speak louder than adjectives, and carry their meaning twice as far.

### A RENT FROM MILITARY PATIENTS?

It is commonly agreed that the War Office grant of a round four shillings per diem for each soldier in a voluntary hospital covers, perhaps, out-of-pocket expenses and no more. Arguments have been urged that the grant should be increased, but most advocates of this have been content to base their case on the still rising cost of provisions and payments to the staff. A Labour representative at Bristol lately put forward a new reason why the War Office grant should be raised. It is one on which we have preferred to meditate before giving it wider publicity. He urged that if Dr. Harrison was right in stating that the grant of four shillings paid for the approximate cost of a soldier's food and drugs at the Bristol General Hospital, then soldiers were in fact being subsidised by charity. To this, as a representative of Labour, he objected, he said. For soldiers should be "fully" provided for by the Government. To this Mr. Herbert Baker replied that even military hospitals had a building provided for them. The argute Mr. Brain's retort was, "They have to pay rent for it." Whereupon Mr. Baker asked, "Do you think you could obtain a rent for us?" Mr. Brain, apparently, was not sure of this, but was convinced that "Someone should move," and that the grant should be big enough to prevent wounded soldiers being in any degree supported by charity. "The objection is more sentimental than vital," replied Mr. Baker, for "charity is not an offensive word." Probably his colleagues at the head of other hospitals will agree. We pause for a reply, and ask, Should hospitals demand from the War Office an addition to the current grant on account of capital expenditure?

### THE ORGANISATION OF EMPLOYERS' SUBSCRIPTIONS.

The collection of working men's subscriptions has now become a science, but hitherto subscriptions from employers have been left to their sense of generosity. The war, however, that convenient name for a multitude of causes, is changing this; and it is right that there should be a change. A cheque for £100 is very useful; but, as we all know, it may not be so generous a contribution as the widow's mite or the workmen's weekly penny. Since employers for the most part have been willing, and even eager, to encourage the organisation of working men's subscriptions, they should hardly complain of applying its principles to themselves. If a weekly subscription to a hospital gives to a workman a sense of friendly, though not of legal, claim, a subscription from an employer is often regarded as a cheap form of insurance. He, too, must learn what his workmen have learnt, to regard it as an obligation of honour and public spirit. We therefore trust that the recent proposal of the committee of the North Staffordshire Infirmary for an annual subscription from each manufacturer in its district on the basis of one shilling per head of every person in his employment will prove as successful as the organisation of workmen's subscriptions have been. We have delayed noticing a most important proposal until such time should elapse as would enable Mr. T. Basil Rhodes, the house-governor, to inform us with his customary courtesy of the progress of the scheme. That time has now arrived. We do not expect impossibilities. But one of the virtues of a well-considered scheme is that it does not flare up and burn out, but grows quietly and steadily.

### SHELL-SHOCKED SOLDIERS IN PLYMOUTH.

THE urgent need for an institution in the West of England where discharged soldiers suffering from shell shock and neurasthenia can be received was emphasised at a meeting of the Plymouth War Pensions Committee on October 4. It was stated that there were a number of cases already in the district for which treatment ought to be provided, but though a place had been opened at Golders Green, and arrangements were proceeding for the establishment of a similar home at Gloucester for the western area, the committee expressed a decided opinion that a rest home, together with land, ought to be provided in the Plymouth locality without delay.

### A FARMERS' HOSPITAL FUND.

WE have read with pleasure the recent statement of the success maintained by the Workpeople's Hospital Fund at York. We read, too, with equal interest Mr. F. H. Thomson's suggestion at Michaelmas that the clergy might be less perfunctory than some have been in recommending from their pulpits the work of the hospital on the Sunday on which, at this season, an offertory is customarily made on behalf of the institution. Mr. Pressley carried the discussion further by making the important suggestion that the clergy should make a new move by uttering a special appeal to the farmers, who, he stated, were doing better now than in the past. We welcome the suggestion because we have already suggested that the hospital's policy should be to organise other funds from other classes, and not be content to allow the workmen's collection to be the principal, if not the sole, organisation to succeed. Whether the clergy are the best people to approach the farmers we leave to the sense of the committee to decide? The point is that each section of the community should have its special fund on the lines of the workmen's collection. In the preceding paragraph we allude to the new scheme for the organisation of employers' subscriptions in North Staffordshire. Here we venture to commend the organisation of a Farmers' Fund. So long as a beginning is made it does not much matter which new class is first approached. But the road to hospital solvency at York can only be traversed with success by the multiplication of sectional funds, through separate organisations, for each different class in the community.

### CUNARD STEAMSHIP COMPANY AND THE WOUNDED.

THE many thousands of wounded soldiers who are located in the hospitals in Liverpool and on the Cheshire side of the Mersey are indebted to the Cunard Steamship Company for entertainment on a right royal scale. During the summer months a series of river trips on one of the company's tenders were made, the party on the last occasion numbering over four hundred. Arrangements are now well in hand for the second season of the winter entertainments for the wounded at the Cunard's palatial new premises on the pier-head at Liverpool. During last season the company entertained five thousand men.

### A SUCCESSFUL MATINEE COMMITTEE.

DURING the ten years in which the Leicester Royal Infirmary Matinée Committee has made its annual efforts to obtain funds, they have been successful in raising £1,105 for the infirmary and £300 for other charities. As in other circumstances, experience has shown the way to developments of organisation, and this year the sum of £250 was aimed at and passed. The incentive to make records each year is due to the generosity of Mr. Oswald Stoll, who places the Palace Theatre, staff, artistes, and orchestra at the disposal of the Committee, and undertakes the cost of printing and advertising, so that every penny taken may be handed over. The Matinée Committee has during the last three years also organised three special matinées for wounded soldiers, and on the present occasion, by the kind thought of a few sympathisers, the whole of the gallery was reserved for them, with the result that several hundred "Tommies" witnessed a very attractive performance. Mr. Arthur Baum is Chairman, Mr. T. H. Prentice, Vice-Chairman, Mr. Geo. Somerville, Treasurer, and Messrs. J. H. Ward and T. Truman Towers, Hon. Secretaries of this useful Committee.

### THE HULL HOSPITAL SUNDAY FUND.

A VERY satisfactory result of the year's work was reported at the annual meeting last week of the Hull Hospital Sunday Fund, which is now in the forty-fifth year of its existence. The total amount collected, £3,033, was by far the largest in the Fund's history. In the special collection made by the Lord Mayor there was recorded a remarkable increase of £860, while advances of £40 and £15 respectively were shown in the collections in places of worship and Sunday schools. There is need of all the money which can be obtained for the Hull hospitals, for the cost per patient has risen to between 28s. and 30s. a week, and it has been found impossible to reduce it. On the motion of Mr. T. R. Ferens, M.P., it was resolved to address an appeal for assistance to Sunday school superintendents and other workers among young people. Sir James Reckitt, always a generous supporter of good causes, mentioned that he was present at the Fund's first meeting over forty-four years ago, and had attended most of the annual meetings since. This is a record of which both Sir James and the Fund may well be proud.

### RAILWAYMEN DEMAND TO CONTROL A HOSPITAL.

The Mexborough Trades and Labour Council, who have lately been showing activity in connection with a number of public questions in their part of the West Riding of Yorkshire, have been urged by their railwaymen members to move for the acquisition, by the trades unions of the district, of the Montagu Hospital; the proposal being for the institution to be completely controlled and financed by the local working-class. Recently this hospital's managing committee urged that the system of a regular subscription-levy already in force among the workpeople of two neighbouring collieries should be extended to all the collieries and works in the hospital area. Excluding military patients, the maintenance of the hospital is estimated at over £2,000 a year, only a portion of which sum is assured. Although Mexborough cannot compare with Crewe, yet railwaymen from Doncaster and elsewhere live there in considerable numbers. Nevertheless, though £2,000 is no great yearly expenditure for a hospital nowadays, the local correspondent of the *Yorkshire Telegraph and Star* does not consider this labour proposal financially practicable. He also contends that "the control and support of the hospital is the inalienable right of everybody in the area which the hospital serves"; this sounds well, but, in the case of such an industrial community, it is not of much value as an argument against control by a trades union.

### BUXTON AND THE CANADIAN SOLDIERS.

The War Office has acquired at Buxton five of the leading hotels, the Buxton Hydro, the Palace Hotel, the Devonshire Hotel, the Grosvenor Private Hotel, and the Pavilion Boarding House, at this famous Derbyshire spa for use as convalescent homes for wounded Canadian officers and men for the duration of the war. The officers are to be accommodated at the Palace Hotel, and the rank and file distributed among the other establishments. The group of homes, conveniently situated within three minutes' walk of each other, will be under the command of Colonel Hanson, of Montreal. It is anticipated that the convalescents, who have previously been under treatment mainly in similar institutions at Ramsgate and other places on the south-east coast, will be in occupation about the middle of this month. It is intended that they shall remain in Buxton until such time as they are regarded as fit to undertake the journey back to Canada. For a considerable period the largest hotel in Buxton, the Empire, has been in use as the Canadian discharge depôt, while another big establishment, the Peak Hydro, is serving as a special Red Cross Hospital for Canadians. Now more than half the total hotel accommodation of the spa will be in the occupation of the Canadians, who thus will form a very considerable proportion of the town's population.

### LONDON'S DUG-OUTS.

The air-raids over London have brought into use many underground places in public buildings and warehouses that have been left for long years unused except for the storing of lumber. It is only natural that people in poor districts, thickly congregated together, should leave their small tenements or large model dwellings and seek a place where they are comparatively secure from any missile. But there is also the graver question as to the effect a stay in these ill-ventilated places may have on the health of the people, especially of babies and young children. A visit to a number of the basements used in the south parts of London show great overcrowding, little or no ventilation, and a thorough lack of any lavatory accommodation. One can better imagine than describe what such a place is like after being inhabited for a length of time with women standing together in close formation with children in their arms. The stench is revolting, and after they have gone it has been found necessary next day thoroughly to disinfect and cleanse the buildings. Happily, it is anticipated that Londoners have seen the worst of these air-raids, and, if that be so, then these unpleasant conditions will soon be over. One striking feature of these public dug-outs is the almost entire absence of anything approaching fright or consternation even under the most trying circumstances. For instance, in one large town hall, where some 1,500 people nightly take shelter, on one occasion when a raid was in progress the electric light suddenly failed. The people remained absolutely quiet until a number of oil lamps were produced from the adjoining works. And this occurred amongst very poor people, who are generally credited with being apt to create a panic in an emergency.

### LEGACIES AND LIABILITIES.

Despite the continual deficit with which they find themselves at the end of each year, it is satisfactory to have to record that the governors of the Worthing Hospital are not getting disheartened in the least—in fact, they have just decided to "carry on" as efficiently as ever, and not to spare expense in any way to the detriment of the work of the institution. To do this they have liquidated the deficit of the past year by drawing on legacies. No criticism can be levelled against them for this action. The money was not wanted for some set or lavish scheme to gratify the whims or fancies of a member of the management committee, but simply to meet the adverse balance-sheet. And these legacies are money left to the institution without condition, and judging from the fact that for the past six years the legacies averaged £1,094 per annum and the deficit £306 per annum, the governors are certainly on the right side of the hedge. But while acting on this new principle every effort must be made to cultivate new subscribers so as to increase the ordinary income of the institution. Worthing Hospital has a splendid record of efficiency and up-to-dateness, thanks to a thoroughly live management committee. In the year just ended there were no fewer than 343 in-patients, 2,037 out-patients, and 533 casualty cases, and it can fairly be asked, Have these 3,000 persons given anything to the institution commensurate with the great benefits received?

### WHAT'S IN A NAME?

The name does not always fit the personality, but in reviewing some of the chief figures of the war one often comes across men who especially possess individualities which match their names. There is, for instance, something knightly about the surname of Stanley, and this characteristic is largely associated with Lord Derby, who has fought so valiantly to bring our fighting forces to their present strength, and with Sir Arthur Stanley, the champion of a large body of women, whose busy lives do not afford leisure for a fight for their own interests. For a Labour Minister, could there be a more appropriate name than Hodge? There is something so sturdy, so dependable, so persistent, so immovable, and so British about the very sound, and these qualities belong in a generous measure to our Minister of Pensions, for whose appointment we may thank the Stockholm Conference. Speaking at Brighton last week the Pensions Minister said that he was engaged in visiting all the centres throughout the country where hospital life and institutional treatment are established, so that he might thoroughly understand all that was being done, not only to help disabled sailors and soldiers by pensions, but to give them something more—a training which would enable them to become even more useful citizens. Such a thorough method of acquiring bed-rock information promises well for the success of the new Minister of Pensions.

### THE MANOR HOUSE HOSPITAL.

The Chief of the Imperial General Staff, Sir William Robertson, emphasised the supreme value of the work carried on at such institutions when he opened recently the Manor House Orthopædic Hospital, charmingly situated between Hampstead and Golders Green. It was impossible, he said, to exaggerate the importance of the hospitals where the wounds of our gallant soldiers were healed, and where also the men were taught trades consistent with their physical capacity and such as would fit them, disabled though they might be, to go out into the world and earn their own livelihood. The Manor House Hospital, which will provide electrical treatment for men discharged from hospital but not completely cured, is equipped with the latest and best appliances.

### THE PAPWORTH HALL SANATORIUM.

Papworth Hall, formerly the residence of Mr. Ernest Terah Hooley, is, as has been already stated in The Hospital, to be utilised as a sanatorium for consumptives. It is proposed to make it the centre of a kind of garden city for tuberculous patients in one of the most healthy and picturesque parts of Cambridgeshire, and open-air treatment and suitable employment will be here provided for soldiers who are suffering from the malady. Dr. Varrier Jones, the tuberculosis officer for Cambridgeshire, is to superintend the sanatorium. Towards the cost of preparing the mansion and the surrounding grounds for the purpose, a gift of £5,000 has been received from Sir Ernest Cassel.

### SHOOTING LIONS FROM A HOSPITAL'S GROUNDS.

The October number of the *London Hospital Gazette* contains many good things and is well up to the high standard of this publication. There is an interesting article on "Some French Army Surgeons of the Past," which is a little spoiled by faulty information in respect of the history of the great French Revolution; the author is wrong in saying that Charlotte Corday was the accomplice of Marat. One of the most interesting papers describes "The Wanderings of an R.A.M.C. Officer," who, in the simplest of language, gives an account of his experiences on several Fronts. The narrative in respect of hospital work in German East Africa covers ground that is new to most readers. Our R.A.M.C. officer thus describes the surroundings of the hospital at Lolkissale: "The hospital consisted of a number of big grass huts and some bell tents, the whole capable of accommodating 400 patients. The hospital was enclosed all round by a border of prickly thorns to keep out the lions and other prowling beasts. Lolkissale was famous for its lions, their roaring at night, coupled with the cries of the hyenas, made sleep impossible until one got used to the din. . . One night we trapped a huge lion over twelve feet long. Now and again parties of us sat up all night in trees in the hope of shooting a lion. The usual plan was to tie up an old ox as bait to a tree near by. . . As the lion made its spring on to the ox we flashed out our acetylene lamps on to the scene, levelled our guns, took careful aim, and fired together if possible." It sounds almost as exciting as air-raids.

### A NOVELTY IN EDITORIALS.

The editorials of the war hospital gazettes are usually the least amusing part of them, unless the conductor of the magazines determines once for all to have done with excuses, apologies, and padding, and uses his space to mention items of personal news that have become crowded out of other columns. A gazette editorial in verse sounds an enormity, yet this feat has been successfully, even gracefully, achieved by the editor of the *Southern Cross* in the latest number to reach us. The remaining contents do not call for detailed comment, except that we notice an attempt to encourage caricature, an art which is always worthy of appreciation, especially in gazettes, which depend for their interest on a concentrated interest in the *personnel* and, as it were, private habits of each hospital.

### SOME BEAMS FROM "THE SEARCHLIGHT."

It was a disappointment. Our pen positively jumped to transcribe a capital jest from the September *Searchlight*, the monthly *Gazette* of the 2nd General Hospital, Manchester, when a splutter was caused by observing the tale to be quoted from a London journal. Of what use is it to quote a lay paper in The Hospital? While a good story is worth quoting it can be quoted too often, and a gazette, with an eye for a good thing, should not be obliged to concern itself with town topics for its witticisms. In another gazette we notice a caricature of the hospital's C.O. in his most expensive uniform at a concert. That is far better—for caricaturist and subject alike. However, the editor of the *Searchlight* has been away convalescing, so we say no more. In his absence a column was lately devoted to "Notes on Gardening," in which we notice the significant phrase "weather permitting" as a condition of carrying out some horticultural operation. The condition is very important, as even people who have no gardens will agree. The contents are agreeably varied: verse, prose, and line pleasantly mixed. There is a good article on being "On Leave," and the whole is lively in tenor.

### THE LIGHTER SIDE OF SERIOUSNESS.

We have remarked before that the *Gazette* of the 3rd London General Hospital has in Miss H. M. Nightingale a contributor worth encouraging. It was a good idea—no matter whose—to give the first place in the October number to an article of hers on the serious side of war. This gay gazette would have marred its claim to cover the whole ground if this subject had always been excluded. The difficulty is, of course, to find the right person to tackle it. Miss Nightingale on the whole has done it well, considering how much she had to omit. She contributes a suggestion: she could not describe the truth. It takes the form of a "Letter to my Goddaughter." There are some jolly photographs in this number, and we note in the articles visible traces of the belief that the end of the war will occur before the next volume of the gazette comes out. We applaud this expectancy. For ourselves we have made it a point of honour to be one of those people who always believe in the autumn that the war will be over in the spring, and always believe in the spring that the war will be finished in the autumn. After all, you have only to wait to see who is proved right. And if you press us with the extravagant charge of having been mistaken, the answer comes pat: We said *the* spring, *the* autumn. The seasons succeed each other, but the spring or the autumn obviously refers to the season in which alone we can rejoice fully—that season, in fine, which sees the end of the war. Miss Nightingale's article has conjured one smile into existence already, as only serious work can. We beg to transfer it to the faces of our readers.

### MRS. ATKINS WRITES HER LETTER.

The sort of correspondence which medical and other Army officers receive from the wives and dependants of soldiers deserves its place in the literature of the war. The following examples are characteristic, and readers can hardly fail to note the crescendo as each succeeding letter is perused. That the letters proceed from different hands is nothing, since Mrs. Atkins, like her gallant husband, is as much a national institution as the immortal "Editor of *Punch*." First—"I have received no pay since my husband has gone nowhere." Second—"Dear Sir, my husband has been at Cristal Palis and has got four days furlong and has now gone away to mind sweepers." Third —"We gave your letter. I am his grandfather and grandmother. He was born and brot up in this house in answer to your letter. Mrs A—— has been put to bed with a little lad, wife of Robert A——." Fourth—"I am writing these few lines to Mrs. Organ who cant write herself and she is expecting to be confined and do wish it." Fifth—"Dear I have been in bed with the doctor for three days. He does not seem to do me any good. If you dont come at once I shall have to send for another." Sixth—"Dear Sir In accordance with your instructions on ring paper I have given birth to a daughter on the 1st." Seventh—"You have changed my little boy into a little girl, will it make any difference." Only Mrs. Atkins can write a letter in which an editor would not alter a single comma, and which the most scrupulous of readers would not wish in any detail to correct.

### THIS WEEK'S DRUG MARKET.

Most of the price changes are again in an upward direction, and in view of the shortage of supplies and persistent demand this tendency is likely to continue. One of the few drugs that are quoted at lower rates is phenacetin, the price of which has dropped materially since last week. The prices of tartaric acid, citric acid, and hexamine are not quite so firmly maintained. Cod-liver oil is offered at very high rates—too high to tempt many purchasers. Cocaine is still available in only very small quantities. An advance in the price of bromides would not come as a surprise, although it is not anticipated that the potassium and sodium salts will be advanced so substantially as was ammonium bromide a few days ago. Clove oil is again dearer; this article seems to advance week by week in sympathy with the rise in the value of cloves. Potassium permanganate is rather dearer, and very little is offered. The scarcity of cream of tartar is unrelieved, and the price is again higher. Bismuth carbonate, sub-nitrate, and salicylate are unchanged at the recent advance.

